# Insisting Pain on the Mid-scapular Line

**DOI:** 10.7759/cureus.6192

**Published:** 2019-11-19

**Authors:** Dimitrios Anyfantakis, Emmanouil K Symvoulakis

**Affiliations:** 1 Primary Care, Primary Health Care Centre of Kissamos, Chania, GRC; 2 Clinic of Social and Family Medicine/Faculty of Medicine, University of Crete, Heraklion, GRC

**Keywords:** multiple myeloma, thoracic pain, early diagnosis

## Abstract

Neoplastic diseases are commonly paired with a wide range of non-specific clinical symptoms. Even the most alarming complaints pose a low positive predictive value making diagnosis of an underlying malignancy a major detective challenge for the primary care physician. Therefore, although cancer may be suspected for not be missed, as management failure within primary care, diagnosis usually occurs in the context of a secondary care setting.

Here we present a case of a patient seeking medical advice from his general practitioner due to a two-week history of back thoracic pain. Following investigations, the patient was early diagnosed with myeloma. Current notion of target-driven laboratory tests utility that may be used as possible clues for the detection of multiple myeloma at a primary care level is also discussed to enhance capacity.

## Introduction

Multiple myeloma is an unusual neoplastic disease mainly affecting the elderly population. Its clinical presentation may be non-specific making timely diagnosis challenging. It has been reported that symptoms of multiple myeloma have a positive predictive value of less than 1%. Primary care physicians, being the first point of contact between the patient and the healthcare system, pose a principal role towards early diagnosis. Timely diagnosis is important in terms of survival and quality of life [1]. In this paper, we present a case of a patient seeking medical advice from his general practitioner due to a two-week history of back thoracic pain.

## Case presentation

A 62-year-old man, former smoker, visited our primary care setting due to a two-week history of thoracic upper right back pain. No improvement after administration of simple analgesics was reported. He was a manual worker with no history of trauma or intake of corticosteroids. His previous medical history was negative for any systemic disease. Routine ultrasound abdominal imaging six months before did not reveal any abnormal findings. Physical examination disclosed marked tenderness upon palpation on the right mid-scapular line.

Performed laboratory tests, slightly on or out of normal limit, were included: calcium 10.2 mg/dl (normal range 8.8-10.2 mg/dl), uric acid 7.3 mg/dl (normal <7 mg/dl) and erythrocyte sedimentation rate (ESR) 35 mm (normal <15 mm). The rest of laboratory investigations were found normal, including complete blood count, urea, creatinine, C-reactive protein, albumin levels and rheumatoid factor.

**Figure 1:**
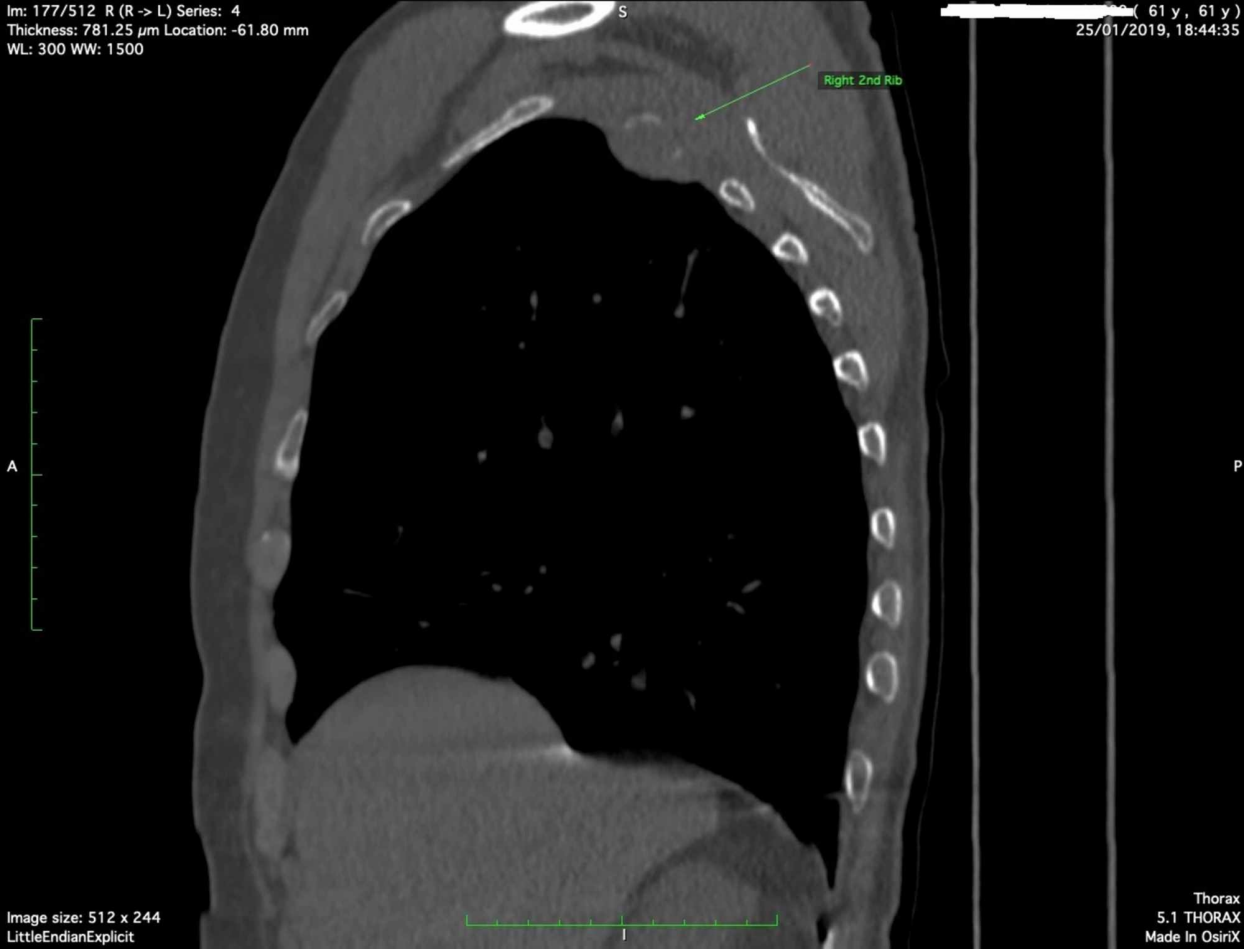
Axial view of CT thorax showing an osteolytic area in the second rib with a surrounding mass

**Figure 2:**
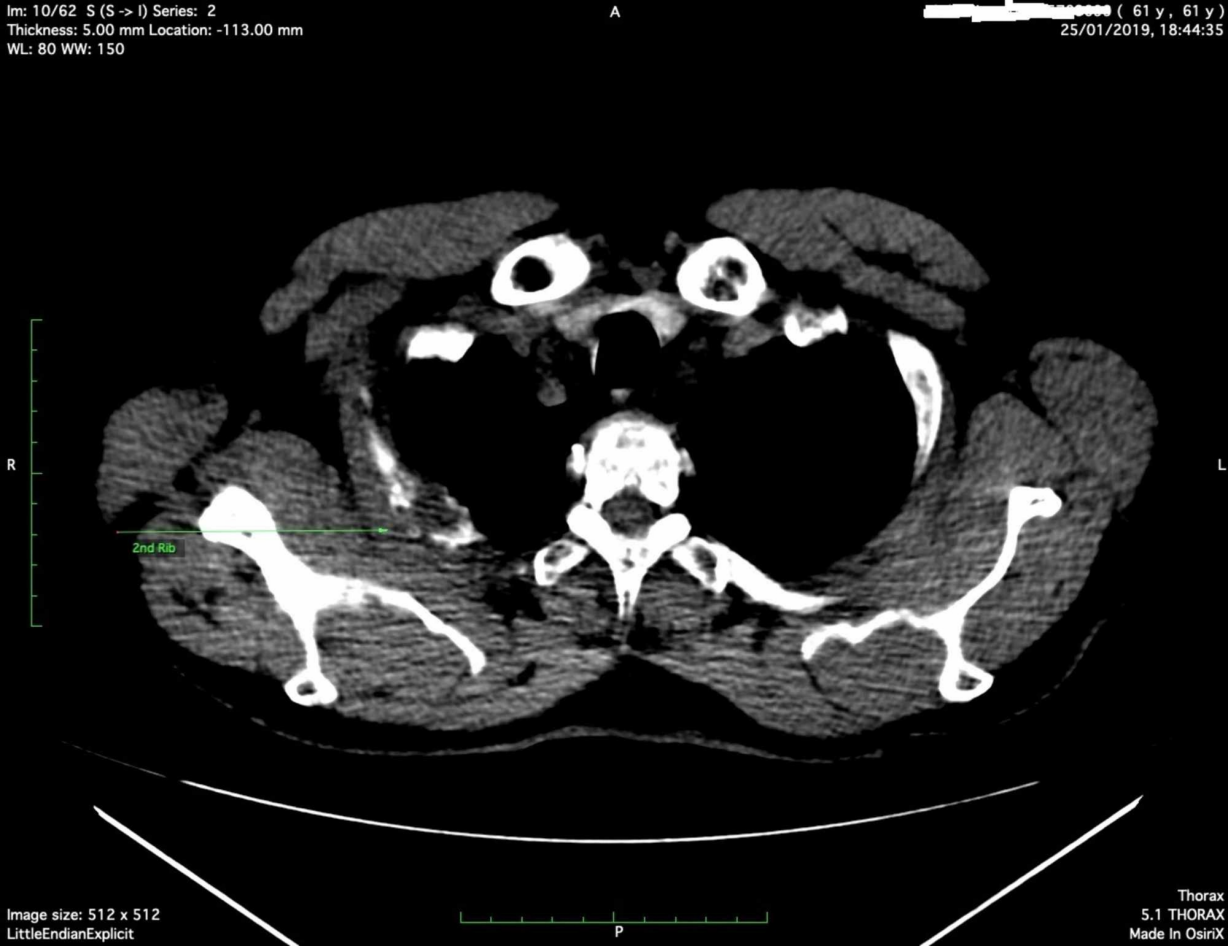
Sagital view of CT thorax showing an osteolytic area in the second rib with a surrounding mass

It was decided to perform a thoracic computed tomography (CT) to investigate the anatomic area of insisting pain. An osteolytic area in the second right rib with a surrounding soft tissue mass was detected (Figure 1, arrow; Figure 2, arrow).

 

## Discussion

Insisting pain at an anatomic point without major mechanical stress susceptibility, an osteolytic rib lesion with a surrounding soft mass and a calcium level of 10.2 mg/dl placed serum and urine protein electrophoresis with immunofixation as an investigational priority among other diagnostic options. Prhotein electrophoresis showed a slight peak in the gamma globulin zone of the serum protein spectrum. Serum free immunoglobulin light chain assay showed light chain type kappa 7.82 mg/l (normal <7.1 mg/l) and light chain type lambda 848 mg/l (normal <3.9 mg/l). Bence Jones albumin was 0.01 mg/l (normal 0.75-4.5 mg/l).

Bone density measurement was not preliminarily performed due to the lack of specific diagnostic utility of this test in a 62-year-old male patient. Prostatic serum antigen (PSA) to exclude prostate malignancy was not indicated due to the negative family history and normal levels of PSA during routine examination a year ago. In the same direction due to the mid-scapular site of pain, the abdominal CT was not a priority. The patient was referred for haematology evaluation on a secondary care centre. Osteomyelitic puncture and biopsy were performed. Pathology confirmed the diagnosis of multiple myeloma. The total time to diagnosis marginally exceeded 15 days.

Multiple myeloma is a haematologic malignancy characterized by significant diagnostic delay (>3 months) due to the low suspicion in the primary care setting [1]. Its clinical and laboratory manifestations usually are not specific [2]. On the time of diagnosis, low haemoglobin [2,3] is considered the most frequent laboratory abnormality followed by hypercalcaemia (calcium level > or =11 mg/dl) and elevated serum creatinine level (>2 mg/dl) [2]. Plasma viscosity and ESR have been also reported as useful inflammatory markers suggesting or excluding the diagnosis in the primary care setting [3]. Additionally, hypercalcaemia and leucopenia when they are combined with clinical symptoms are also suggestive for myeloma diagnosis [4]. The acronym ‘CRAB’ (hypercalcaemia, renal impairment, anaemia and bony lesions) has been reported as a helpful mnemonic tool for the general practitioner that should trigger the diagnostic suspicion [5].

Initial diagnostic evaluation requires careful medical history, physical examination and laboratory work-up of renal function, haemoglobin and calcium levels, serum and urine protein electrophoresis [6]. Active disease is important to be diagnosed immediately and cured, while asymptomatic disease requires only a short clinical follow-up [6].

In this case, the mentioned blood neoplastic disease was not clinically much evident. Unspecific laboratory tests were normal. The diagnostic suspicion for multiple myeloma was triggered in terms of ‘deduction’-driven likelihood. Multiple myeloma appeared in this primary care patient as the most probable, and therefore specific laboratory work-up was commenced in order to confirm or rule out the diagnostic hypothesis.

Interestingly, current clinical data support that in the primary care the only cancer markers that may contribute towards a timely diagnosis of a neoplastic disease are CA125 and PSA [7].

## Conclusions

Primary care physicians should include multiple myeloma in the differential diagnosis of middle aged patients, by fully investigating non-specific but insisting musculoskeletal symptoms, and trying to close gaps between clinical manifestations and routine laboratory findings by not underestimating even upper normal limit deviations of serum calcium.
